# Clinical validation of an AI-based automatic quantification tool for lung lobes in SPECT/CT

**DOI:** 10.1186/s40658-023-00578-z

**Published:** 2023-09-21

**Authors:** Emilie Verrecchia-Ramos, Olivier Morel, Merwan Ginet, Paul Retif, Sinan Ben Mahmoud

**Affiliations:** 1 Department of Medical Physics, Mercy Hospital, CHR Metz-Thionville, 1, Allée du Château, 57530 Ars-Laquenexy, France; 2 Department of Nuclear Medicine, Mercy Hospital, CHR Metz-Thionville, 1, Allée du Château, 57530 Ars-Laquenexy, France; 3 CNRS, CRAN, Université de Lorraine, 54000 Nancy, France

**Keywords:** Lobar quantification, Perfusion SPECT/CT, Ventilation SPECT/CT, AI-based segmentation

## Abstract

**Background:**

Lung lobar ventilation and perfusion (V/Q) quantification is generally obtained by generating planar scintigraphy images and then imposing three equally sized regions of interest on the data of each lung. This method is fast but not as accurate as SPECT/CT imaging, which provides three-dimensional data and therefore allows more precise lobar quantification. However, the manual delineation of each lobe is time-consuming, which makes SPECT/CT incompatible with the clinical workflow for V/Q estimation. An alternative may be to use artificial intelligence-based auto-segmentation tools such as AutoLung3D (Siemens Healthineers, Knoxville, USA), which automatically delineate the lung lobes on the CT data acquired with the SPECT data. The present study assessed the clinical validity of this approach relative to planar scintigraphy and manual quantification in SPECT/CT.

**Methods:**

The Autolung3D software was tested on the retrospective SPECT/CT data of 43 patients who underwent V/Q scintigraphy with ^99m^Tc-macroaggregated albumin and ^99m^Tc-labeled aerosol. It was compared to planar scintigraphy and SPECT/CT using the manual quantification method in terms of relative lobar V/Q quantification values and interobserver variability.

**Results:**

The three methods provided similar V/Q estimates for the left lung lobes and total lungs. However, compared to the manual SPECT/CT method, planar scintigraphy yielded significantly higher estimates for the middle right lobe and significantly lower estimates for the superior and inferior right lobes. The estimates of the manual and automated SPECT/CT methods were similar. However, the post-processing time in the automated method was approximately 5 min compared to 2 h for the manual method. Moreover, the automated method associated with a drastic reduction in interobserver variability: Its maximal relative standard deviation was only 5%, compared to 23% for planar scintigraphy and 19% for the manual SPECT/CT method.

**Conclusions:**

This study validated the AutoLung3D software for general clinical use since it rapidly provides accurate lobar quantification in V/Q scans with markedly less interobserver variability than planar scintigraphy or the manual SPECT/CT method.

## Background

Determining regional lung ventilation and perfusion (V/Q) reveals pulmonary function and can help guide lung resection decisions [1]. Recently, the need for lung segmentation has increased due to the development of new treatments for emphysema such as endobronchial valves [2, 3]: V/Q estimations are needed in such cases to identify the ideal target lobe and lung [4, 5]. A common way to estimate V/Q is to conduct planar scintigraphy, which employs a very simple method for quantifying the counts in the lungs; specifically, the left and right lungs on planar anterior and posterior scintigraphy images are divided into equal thirds by applying standard and equally sized rectangular regions of interest (ROIs) [6]. However, this approach does not accurately reflect true lobar anatomy [7]. In fact, it requires the division of the left lung into three parts despite the fact that it bears only two lobes; as a result, the counts in the middle part must be halved and each half then added to the counts in the inferior and superior lobes. By contrast, 3D SPECT/CT scanning permits a more precise definition of the lobar volume and integrates attenuation correction, thus resulting in more accurate lobar quantification [8]. However, it can be very time-consuming to manually delineate each lobe on the CT dataset, which means that this approach is not suitable for use in the routine clinical workflow.

To improve this, artificial intelligence (AI) and the latest deep-learning techniques have been used to automatically segment the lung lobes on the CT data of SPECT/CT lung scans, thus quickly providing V/Q estimates. Theoretically, such methods should provide more precise and accurate estimates than the conventional planar estimation but within a similar time frame. Indeed, evaluations of some of these algorithms (i.e., the HERMES hybrid 3D lung lobe quantification algorithm from Hermes Medical Solutions and the adaptive iterative reconstruction algorithm from GE Healthcare) show they are sufficiently robust for clinical application and improve lobar quantitation relative to the conventional planar method [7, 9].

Another AI-based auto-segmentation algorithm is AutoLung3D, which was developed by Siemens Healthineers (Knoxville, USA). To validate the usefulness of AutoLung3D for lung lobe segmentation in lung SPECT/CT and estimations of V/Q, we conducted the present investigation. Specifically, we compared this method to planar estimations and manual segmentation of the SPECT/CT data in terms of accuracy and interobserver variability. To our knowledge, this study is among the first to directly compare planar, manual, and automated methods in terms of V/Q estimate accuracy and interobserver variability.

## Methods

### Patients

In total, 43 consecutive patients who underwent routine V/Q scintigraphy with SPECT/CT acquisitions in September–October 2021 to determine the relative lobar V/Q were identified retrospectively by reviewing the medical records. This study period was chosen randomly; therefore, the study cohort was typical of our clinical practice population. The average age of the patients was 66 (range, 20–94) years, 56% were female, and 49% had emphysema.

### SPECT-CT imaging

All V/Q scintigraphy scans were performed with a hybrid SPECT/CT scanner (Symbia T16, Siemens Healthineers, Knoxville, USA). Ventilation scintigraphy was performed after inhalation of approximately 60 MBq of an ultra-fine dispersion ^99m^Tc-labeled carbon (Technegas, Cyclomedica, Dublin, Ireland). Perfusion scintigraphy was performed after intravenously injecting 235 (range, 210–257) MBq of ^99m^Tc-macroaggregated albumin (Pulmocis, CIS Bio International, Gif-sur-Yvette, France). SPECT imaging was performed over 360° (140 keV ± 7.5%, scatter window 110–130 keV for dual-energy window scatter correction, matrix 128 × 128, zoom 1.45, 64 projections at steps of 5.6°, and acquisition times of 15 s per step for ventilation and 10 s per step for perfusion) in a single-bed position, with a low-energy high-resolution (LEHR) collimator according to the national recommendations [10]. This kind of collimator, which is the most common collimator used in France, provides good spatial resolution, but its sensitivity is half that of a general-purpose collimator [11]. Therefore, the administered activities are twice those recommended in the international guidelines, which specifically apply to general-purpose collimators [12]. To correct the SPECT images for attenuation, a free-breathing CT acquisition was also performed using the following parameters: X-ray tube voltage = 110 kV, the X-ray tube current modulated to compensate for patient attenuation (CareDose mAs ref = 110 mAs), pitch = 0.8, and collimation = 16 × 1.2 mm. The CT data used for lung segmentation were reconstructed with the sharp lung kernel (B70s) and a slice thickness of 1 mm.

The SPECT reconstruction was performed with an iterative algorithm (proprietary algorithm Flash3D: 3D ordered-subset expectation maximization) using four iterations and eight subsets, collimator–detector response compensation, scatter compensation, and attenuation correction driven by the CT data. A Gaussian post-filtration of 5 mm FWHM was applied to improve the signal-to-noise ratio. The final voxel size in the reconstructed SPECT images was 3.3 mm × 3.3 mm × 3.3 mm.

### Planar imaging

Planar anterior and posterior images were generated by forward projection of the SPECT images at appropriate angles [13] using an in-house Python script. To mimic attenuation, the Hounsfield units measured in the CT images were converted into a µ-map showing the 140 keV ^99m^Tc gamma-ray attenuation coefficient value at each point (*x*, *y*, *z*) of the emission volume. Subsequently, the reconstructed emission images were projected through the µ-map, namely with an exponential attenuation of the integral of µ. This method allowed us to produce high-count planar images that were comparable to those obtained by a traditional planar acquisition [14].

### V/Q quantification

**Fig. 1:**
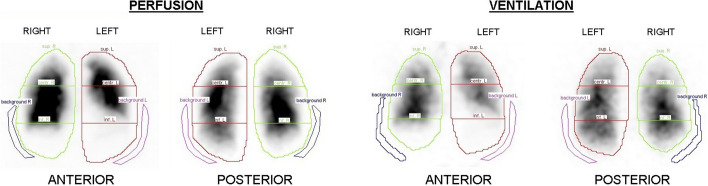
Lung segmentation on the planar scintigraphy data for perfusion and ventilation. Each lung was divided into three regions of interest by using a template that imposes three rectangular equally sized areas. Representative data of a single patient are shown

The planar ventilation and perfusion images were analyzed according to the current clinical standards. Specifically, after a manual delineation of the lungs, a template composed of six rectangular, equally sized regions was applied to divide each lung into three ROIs. Finally, the background counts, which were measured in specific external ROIs, were subtracted from the counts measured in each lung ROI. An example of the ROIs defined on planar V/Q scintigraphy is shown in Fig. 1. For the right lung, each third of the lung was considered to be the corresponding lobe (superior, middle, and inferior). By contrast, given that the left lung does not contain a middle lobe anatomically, the central ROI counts of the left lobe were halved and each half was added to the superior and inferior left lobe counts.

**Fig. 2:**
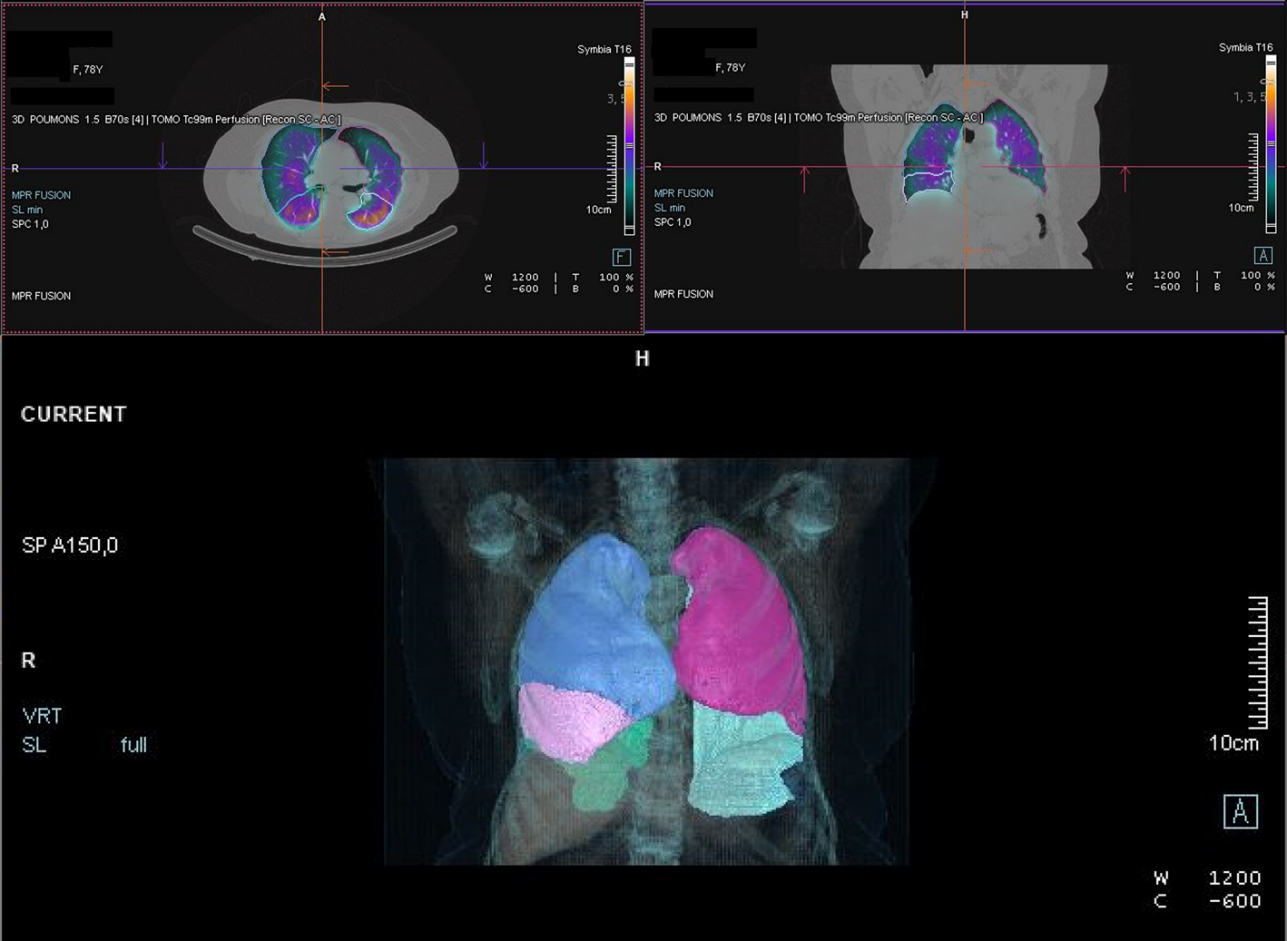
Automated lobar segmentation on the perfusion SPECT/CT dataset using the AI-based algorithm AutoLung3D. Representative data of a single patient (the same patient in Fig. 1) are shown

**Fig. 3:**
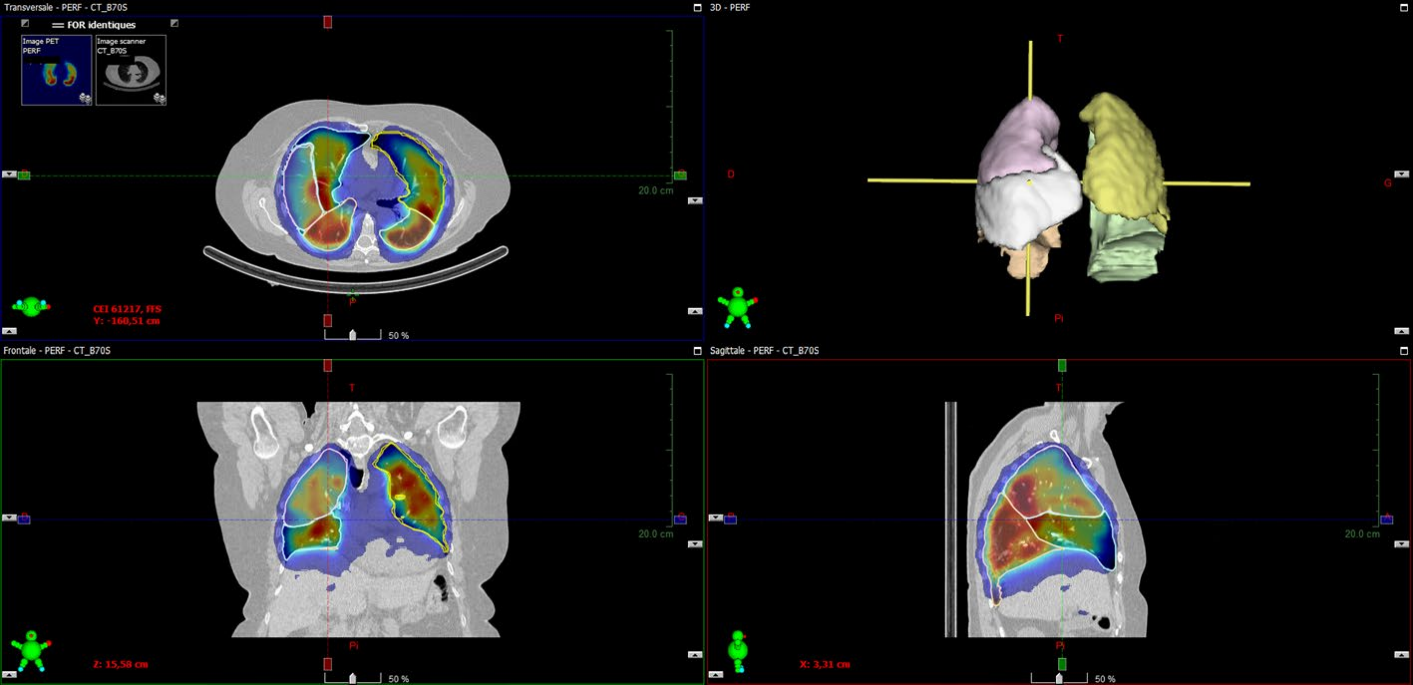
Manual lobar segmentation on the perfusion SPECT/CT dataset imported in Eclipse. The segmentation was conducted by an experienced nuclear physician. Representative data of a single patient (the same patient in Figs. 1 and 2) are shown

The lung segmentation in the V/Q SPECT/CT datasets was performed in two ways, namely an automated way and a manual way. The automated method was conducted with AutoLung3D, which is the AI-based supervised deep-learning tool developed by Siemens. An example of AutoLung3D segmentation is shown in Fig. 2. These data came from the same patient whose planar scintigraphy data are shown in Fig. 1. The manual method, which can serve as the reference method because it precisely describes the actual fixation in each lobe, involved manual segmentation of each lobe by an experienced nuclear physician. To facilitate this segmentation, we developed a home-made Python routine to convert the SPECT/CT data into a DICOM format that could be read by the contouring-dedicated module of the treatment planning system Eclipse (Varian Medical Systems, Palo Alto, California, USA). This reflects the fact that Eclipse is not developed to work with SPECT images; consequently, these images must be converted into the fictive PET modality in the DICOM header. Our method also involved creating one DICOM file per slice to mimic PET files, whereas all slices are initially gathered in the same DICOM file in the SPECT images. Once the lobar segmentation was performed manually on the CT dataset in Eclipse, the structures were copied to the V/Q SPECT datasets and the counts inside each lobe were determined. An example of this manual segmentation performed in Eclipse is shown in Fig. 3. Again, these data came from the same patient whose planar and automated data are shown in Figs. 1 and 2.

### Interobserver variability

The interobserver variability of each quantification method was determined with a subset of ten randomly selected patients. For this, three experienced nuclear physicians conducted the same V/Q quantifications independently.

### Statistical analyses

For each of the 43 patients, the relative distribution (%) of the radioactive tracer in each lobe in the left and right lung was obtained with the three quantification methods. These data were expressed as mean and 95% confidence intervals and ranges. The relative distributions in each total lung were also expressed in the same way. The three methods were compared in terms of the relative distributions in each lobe and each total lung by using a one-way analysis of variance (ANOVA) employing a Welch’s test with an alpha risk *α* = 5%. These analyses were conducted separately for the ventilation and perfusion datasets. The interobserver variability data of each method were presented as the relative standard deviation of the results [15].

## Results

**Fig. 4:**
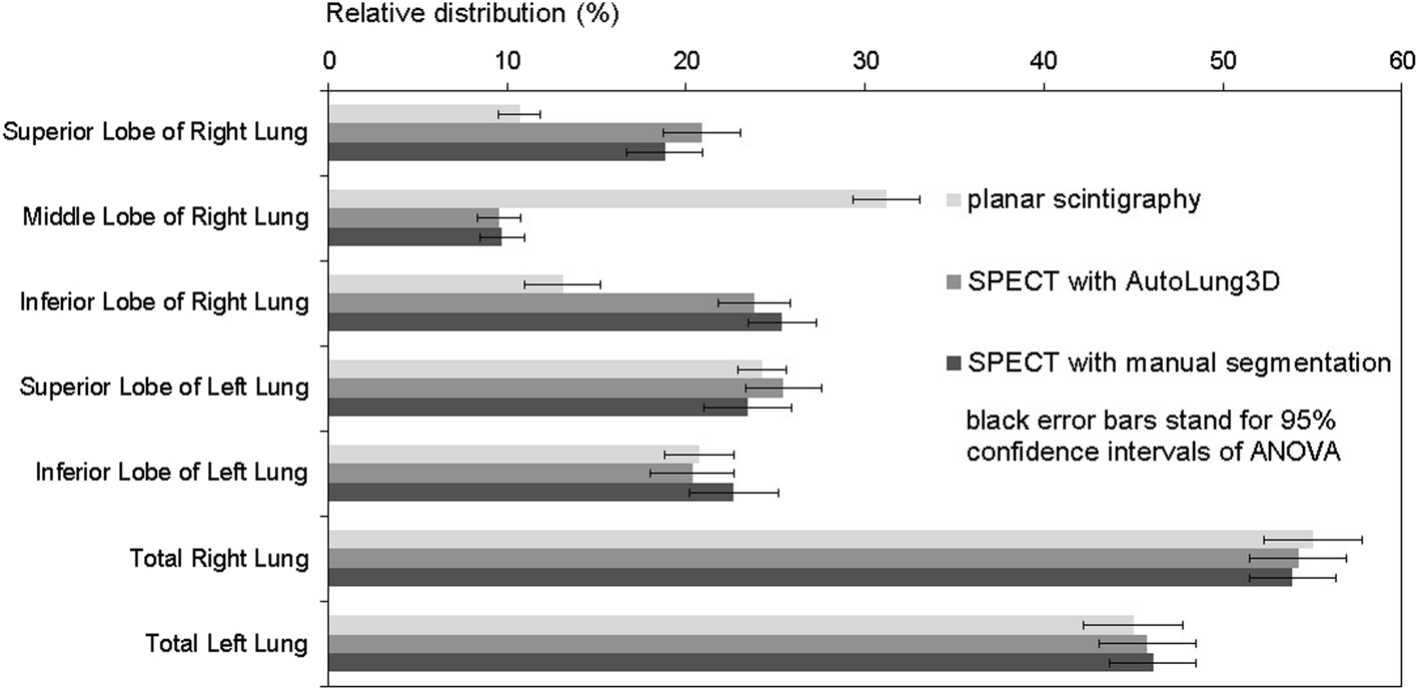
Comparison of the three quantification methods in terms of relative ^99m^Tc-labeled aerosol distribution (%) during lung ventilation analysis. *p < 0.05 for the indicated lobe, as determined by Welch’s test-based ANOVA. Significant differences between the methods can be seen by viewing the 95% confidence intervals: If two methods do not show overlap of these bars, they differ significantly in relative distribution

**Fig. 5:**
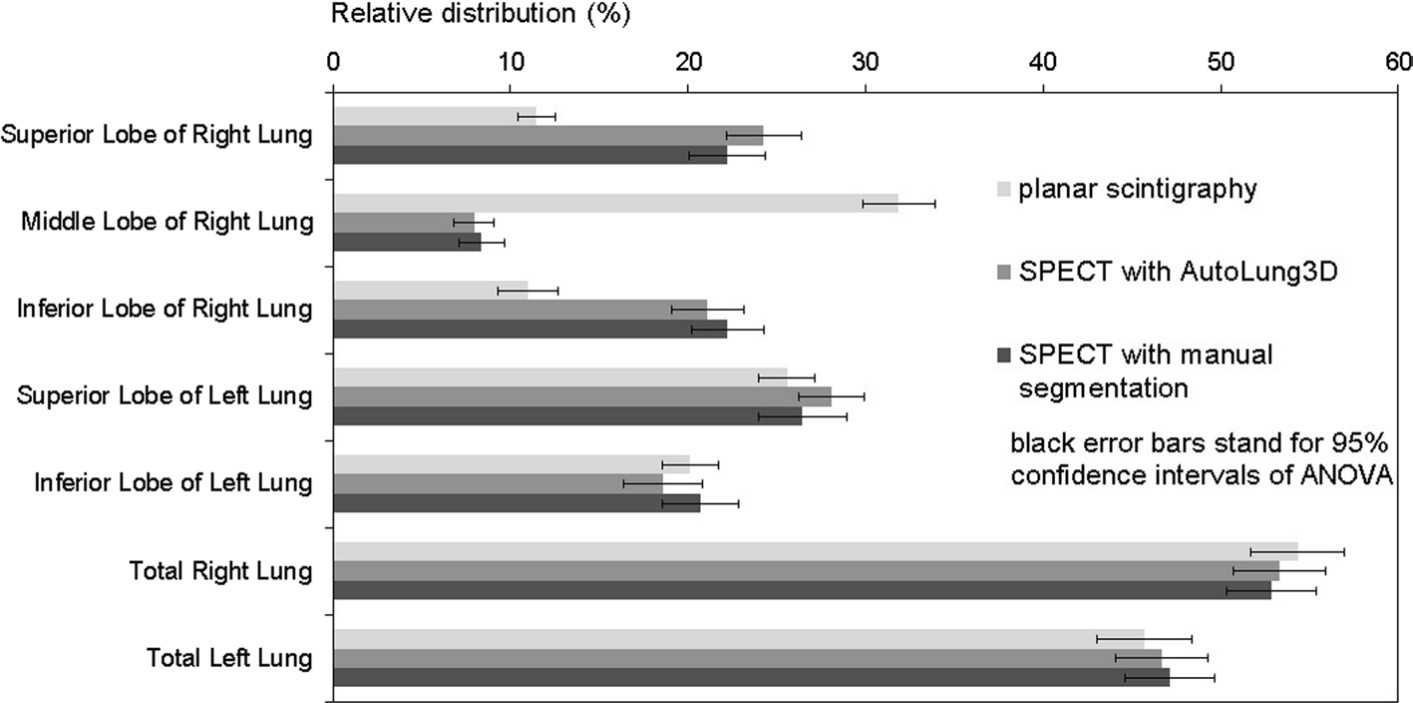
Comparison of the three quantification methods in terms of relative ^99m^Tc-macroaggregated albumin distribution (%) during lung perfusion analysis. *p < 0.05 for the indicated lobe, as determined by Welch’s test-based ANOVA. Significant differences between the methods can be seen by viewing the 95% confidence intervals: If two methods do not show overlap of these bars, they differ significantly in relative distribution

Representative segmentations are shown for one patient in Figs. 1, 2, and 3. The relative radiotracer distribution data that were produced by the planar, automated, and manual quantification methods to estimate V/Q are summarized in Tables 1 and 2, respectively. They are also presented graphically in Figs. 4 and 5, respectively. These data show that while the three methods did not differ in terms of any left lung lobe or total lung estimates, the planar scintigraphy method differed from the automatic and manual SPECT/CT methods in terms of the right lung lobe estimates. Thus, the ventilation data showed that the planar method detected lower relative distributions in the superior (10.7% vs. 20.9% and 18.8%; *p* < 0.001) and inferior right (13.1% vs. 23.8% and 25.4%; *p* < 0.001) lobes and higher relative distributions in the middle right lobe (31.2% vs. 9.5% and 9.7%; *p* < 0.001). The perfusion data showed very similar trends. By contrast, the automated and manual SPECT methods yielded similar results for all lobes and lungs for ventilation (Table 1 and Fig. 4) and perfusion (Table 2 and Fig. 5). This was also true for the 21 patients with lung parenchymal changes due to emphysema. Although these changes tended to obscure the fissures on the CT dataset and therefore required even more time-consuming manual delineation, they had little impact on the Autolung3D results (Table 3). Thus, automated segmentation remains accurate in such cases. However, the AutoLung3D software could not manage a patient who had had a lobectomy before imaging: It produced a five-lobe segmentation and did not detect the missing lobe. This deficiency may reflect the training dataset used in the deep-learning process, which probably consisted of patients who had not undergone a lobectomy. Although lobectomy cases are rare in clinical practice, our finding suggests that AutoLung3D cannot accurately manage cases of gross anatomic change and should not be used in such cases.

**Table 1:** Average [range] relative distribution (%) of the ventilation radiotracer in the right and left lung lobes and total lungs, as determined by using the three quantification methods

Quantification method	Right lung lobe values			Left lung lobe values		Total lung values	
	Superior lobe	Middle lobe	Inferior lobe	Superior lobe	Inferior lobe	Right lung	Left lung
Planar scintigraphy	10.7 [2.4–21.6]^a^	31.2 [8.5–40.5]^a^	13.1 [1.2–34.1]^a^	24.3 [10.9–39.2]	20.7 [10.2–47.6]	55.0 [13.3–78.8]	45.0 [21.2–86.7]
SPECT AutoLung3D	20.9 [6.0–38.0]	9.5 [1.0–19.0]	23.8 [6.0–43.0]	25.5 [15.0–53.0]	20.3 [3.0–34.0]	54.2 [13.0–79.0]	45.8 [21.0–87.0]
SPECT manual segmentation	18.8 [4.7–39.3]	9.7 [1.6–21.2]	25.4 [9.3–45.1]	23.4 [0^b^–48.9]	22.7 [2.1–44.3^b^]	53.9 [21.7–78.3]	46.1 [21.7–78.3]
p*	** < 0.001**	** < 0.001**	** < 0.001**	0.433	0.343	0.824	0.828

Bold *p*-values mean a statistically significant difference between the quantification values obtained with the manual and automated methods

The data are shown as mean [range]

^a^The automated and manual values both differ significantly from the planar scintigraphy value

^b^One patient had had a lobectomy, which was taken in account only on manual segmentation as it was not visible on planar images and the lobectomized lung was not accurately segmented by the AutoLung3D algorithm

*Determined by ANOVA

**Table 2:** Average [range] relative distribution (%) of the perfusion radiotracer in the right and left lung lobes and total lungs, as determined by using the three quantification methods

Quantification method	Right lung lobe values			Left lung lobe values		Total lung values	
	Superior lobe	Middle lobe	Inferior lobe	Superior lobe	Inferior lobe	Right lung	Left lung
Planar scintigraphy	11.5 [4.6–21.0]^a^	31.9 [13.5–45.6]^a^	11.0 [0.5–28.2]^a^	25.6 [15.2–41.7]	20.1 [10.1–36.0]	54.3 [22.4–71.1]	45.7 [28.9–77.6]
SPECT AutoLung3D	24.3 [7.0–43.0]	8.0 [1.0–15.0]	21.1 [8.0–36.0]	28.1 [14.0–42.0]	18.6 [4.0–38.0]	53.3 [20.0–69.0]	46.7 [31.0–80.0]
SPECT manual segmentation	22.2 [5.3–43.9]	8.4 [1.6–20.3]	22.2 [7.3–37.0]	26.5 [0^b^–45.3]	20.7 [4.1–33.9^b^]	52.8 [23.0–69.6]	47.2 [30.4–77.0]
p*	** < 0.001**	** < 0.001**	** < 0.001**	0.121	0.374	0.712	0.718

Bold *p*-values mean a statistically significant difference between the quantification values obtained with the manual and automated methods

The data are shown as mean [range]

^a^The automated and manual values both differ significantly from the planar scintigraphy value

^b^One patient had had a lobectomy, which was taken in account only on manual segmentation as it was not visible on planar images and the lobectomized lung was not accurately segmented by the AutoLung3D algorithm

*Determined by ANOVA

**Table 3:** Average relative distribution (%) of the radiotracer, as determined on SPECT-CT images by manual segmentation and AutoLung3D, for the 21 patients with emphysema

Quantification method	Right lung lobe values			Left lung lobe values		Total lung values	
	Superior lobe	Middle lobe	Inferior lobe	Superior lobe	Inferior lobe	Right lung	Left lung
Ventilation SPECT AutoLung3D	17.7	9.2	25.5	22.7	24.9	52.4	47.6
Ventilation SPECT manual segmentation	20.3	8.3	23.8	26.3	21.2	52.4	47.6
Error (%) on ventilation distribution	− 2.6	+ 0.9	+ 1.7	− 3.6	− 3.7	0	0
Perfusion SPECT AutoLung3D	21.1	8.3	22.1	26.6	21.8	51.5	48.5
Perfusion SPECT manual segmentation	24.1	7.3	20.7	29.3	18.6	52.1	47.9
Error (%) on perfusion distribution	− 3.0	+ 1.0	+ 1.4	− 2.7	+ 3.3	− 0.6	+ 0.6

**Fig. 6:**
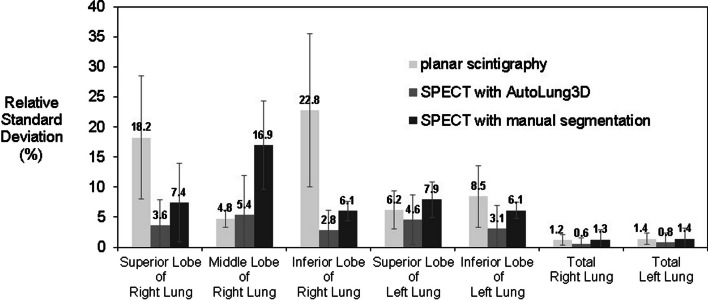
Interobserver variability of the three methods. Ventilation quantifications were performed by three experienced nuclear physicians with planar, manual, and automated segmentation in ten patients. The data are expressed as average relative standard deviation (%) of the relative distribution in each lobe/lung. The gray error bars indicate the standard deviation of the ten patients

**Fig. 7:**
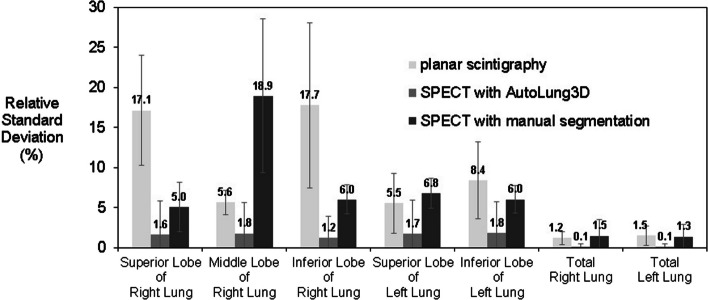
Interobserver variability of the three methods. Perfusion quantifications were performed by three experienced nuclear physicians with planar, manual, and automated segmentation in ten patients. The data are expressed as average relative standard deviation (%) of the relative distribution in each lobe/lung. The gray error bars indicate the standard deviation of the ten patients

Figures 6 and 7, respectively, summarize the V/Q quantification interoperator variability associated with the three quantification methods. In general, the planar method demonstrated the greatest variability in lobe estimates, particularly the inferior right lobe: The greatest average relative standard deviation in all estimates was 22.8% (vs. 2.8–6.1% for the other methods). The exception was the middle right lobe; here, the manual method showed the highest variability (greatest average relative standard deviation = 18.9% vs. 5.6% for the planar method and 5.4% for the automated method). This result may be explained by the horizontal fissure that delimitates the middle right lobe, which can be very difficult to distinguish on the CT dataset in some cases (emphysema for instance). The planar and manual methods were associated with similar variability in the whole lungs. The automated method consistently showed the least variability of all three methods, including in the whole lungs; indeed, the greatest average relative standard deviation in all automated measurements was 5.4% (vs. 22.8% for the planar method and 18.9% for the manual method).

## Discussion

Clinical V/Q estimates are generally obtained by conventional planar scintigraphy and segmentation of the lungs generated by imposing a six-ROI template on the images. This approach is both practical and fast. An alternative approach is to manually segment each lobe of each lung on the CT dataset, copy these segmentations on the SPECT ventilation or perfusion images, and then calculate the relative distribution values. This approach is much more laborious and time-consuming than the planar method because before export to the SPECT dataset, multiple steps must be performed: (1) the SPECT data have to be converted into a format that can be read by Eclipse, (2) the volumes of interest (lungs, lobes, and fissures) have to be created, (3) the fissures have to be segmented by the nuclear physicians, and (4) the lobe structures must undergo Boolean extraction. These steps together take approximately 2 h for each patient, which is incompatible with a clinical workflow. Nonetheless, this manual approach precisely describes the actual fixation in each lobe and was therefore considered the reference method in this study.

This study showed that while the planar and manual estimates for the whole lungs and the left lung lobes were quite similar, marked differences were observed for all three lobes in the right lung. Specifically, compared to the manual estimates, the planar estimates were much lower for the inferior and superior right lobes and much higher for the middle lobe. These results are in line with previous studies; specifically, marked right-lobe differences were observed between planar and SPECT/CT V/Q quantifications when manual segmentation [8] or an equivalent semi-automated lung lobe segmentation software (Hermes Lung Lobe Quantification; Hermes Medical Solutions, Stockholm, Sweden [7]) was employed. Our study significantly expands this field by directly comparing the planar, manual SPECT/CT, and semi-automated quantifications. A similar methodology was used by Genseke et al. [9] to validate the contender semi-automated segmentation tool Q. Lung (GE Healthcare, Haifa, Israel); they reported similar results, namely a great difference between planar and SPECT quantifications and good agreement between manual and automated segmentations on the SPECT/CT. They also demonstrated a strong interrater agreement for the semi-automated method but did not compare it to planar or manual SPECT delineations. By contrast, the current study compared the three quantification methods in terms of interoperator variability and showed that the semi-automated technique was particularly robust in this property.

The difference between planar and SPECT/CT quantifications can be explained by the right horizontal and oblique fissure positions: Since the conventional planar estimation involves dividing the right lung into three equal thirds, these positions are not taken into account. By contrast, the SPECT/CT data allow each lobe to be precisely segmented by following these fissures, which are visible on the CT dataset. Thus, the manual method provides a much more accurate estimation of the actual tracer distribution. Moreover, planar images involve the overlap of anatomic segments [16] and the superimposition of detected counts degrades the quantification. The fact that the planar and manual methods did not differ markedly in terms of V/Q estimates for the left lung lobes reflects the simpler anatomy of the left lung. Moreover, the V/Q estimates for the total lungs were equivalent for the planar and manual methods for a related reason; namely, the anatomic details no longer play an important role in these estimates. Thus, compared to planar scintigraphy, 3D SPECT/CT provides important information about the actual anatomy of the right lung and therefore allows the right lung lobes to be more precisely quantified. Moreover, the CT-based segmentation is not biased by the radiotracer distribution in the lungs, which is often very nonhomogeneous in patients with respiratory disease. For example, patients with severe airway obstruction can demonstrate hotspots on ventilation scintigraphy. Nonetheless, such hotspots will not impair CT-based segmentation. Notably, we did not subtract the hotspots from the images in the present study; consequently, such hotspots had an identical impact on all three quantification methods.

Given these advantages of manual quantification but its time-consuming nature, an automated approach is needed. To address this, we tested the AI-based automated AutoLung3D algorithm. We observed that the V/Q estimates of this automated approach were similar to those determined by the manual method for all lobes and both total lungs, including the right lung lobes. Moreover, the automated approach reduced the post-processing time from 2 h to approximately 5 min; this included the segmentation check by the physician. In addition, our study showed that the automated approach had a further advantage over the manual method: It dramatically reduced interobserver variability from a maximal average relative standard deviation of 18.9% with the manual method to only 5.4%.

This study had five main limitations. First, the SPECT V/Q estimations were based on a free-breathing CT dataset, which may increase uncertainty regarding the lung and lobe segmentations. A respiratory-gated CT would allow more precise segmentations.

Second, the angular sampling of 5.6° chosen for SPECT acquisitions is debatable. A lower angular step would theoretically decrease the streak artifacts in the reconstructed image [17]. However, this kind of artifact is strongly reduced with iterative reconstructions [18]. In practice, we did not observe any difference on the OSEM reconstructed lung images, between 128 projections/2.8° acquisitions and 64 projections/5.6° step acquisitions.

The third limitation was the sample size (*n* = 43), relatively small for a clinical validation study. This reflects the time-consuming nature of the manual delineation (~ 2 h/patient). Nonetheless, the sample size was sufficient to identify statistically significant differences between planar and SPECT quantifications and to show that the quantification values are very close between manual and Autolung SPECT delineations. Moreover, our sample size exceeds those used to compare planar scintigraphy with manual SPECT/CT (*n* = 17 [8]) or to validate the Hermes (*n* = 30 [7]) or Q. Lung (*n* = 39 [9]) tools mentioned above.

The fourth limitation was that we only analyzed a single patient with gross structural changes, namely those due to lobectomy. This reflected the fact that the patients were randomly selected to represent our clinical practice population. We will assess the accuracy of the AutoLung3D software in such cases in a separate study. Nonetheless, it should be noted that our study population also included 21 patients with lung parenchymal changes (e.g., emphysema); which did not impact the Autolung3D results. Thus, automated segmentation remains accurate in such cases.

The fifth and final limitation of this study is that is does not assess the robustness of the AI method in relation to various SPECT/CT scanners and their respective settings. Undoubtedly, the robustness of the AI method is correlated with the similarity between the images in the user’s dataset and those employed to train the deep-learning algorithm. As manufacturers do not provide information concerning the composition of the training dataset, it is advisable to conduct a robustness evaluation for each individual SPECT/CT device and its unique acquisition and reconstruction settings. Nevertheless, given that these settings are optimized for the specific clinical task of lung CT imaging, substantial variations in image quality across different SPECT/CT scanners are not to be expected. Under these circumstances, the findings of this study are anticipated to be applicable across a range of devices and institutional settings.

## Conclusions

Our study suggests that AutoLung3D may be a feasible alternative to planar scintigraphy for routine clinical V/Q estimations because of three significant advantages, as follows. First, because it is based on SPECT/CT 3D datasets, it provides accurate and realistic estimations of the relative distributions in each lung lobe. Second, it is rapid; this makes SPECT/CT usable in the clinical routine, which was not possible previously. Third, its semi-automated AI-based component greatly reduces interobserver variability, which is a guarantee of quality in patient care. Indeed, because of these advantages, this tool is now often used in our nuclear medicine department to provide relative lobar quantification in V/Q SPECT/CT. Nevertheless, users of AutoLung3D should be aware that it may not be useful in patients with gross anatomic changes to their lungs such as lobectomy.

## Data Availability

The datasets used and/or analyzed during the current study are available from the corresponding author on reasonable request.

## Abbreviations

AI: Artificial intelligence
ANOVA: Analysis of variance
CT: Computed tomography
LEHR: Low energy high resolution
ROI: Region of interest
SPECT: Single-photon emission computed tomography
V/Q: Ventilation and perfusion
3D: Three dimensional
